# Usefulness of CT-scan in the diagnosis and therapeutic approach of gallstone ileus: report of two surgically treated cases

**DOI:** 10.1186/1471-2482-13-S2-S6

**Published:** 2013-10-08

**Authors:** Danzi Michele, Grimaldi Luciano, Fabozzi Massimiliano, Reggio Stefano, Danzi Roberta, Soscia Ernesto, Amato Bruno

**Affiliations:** 1 Department of Specialized Surgery - Division of Gastrointestinal Surgery Rehabilitation of Election and Emergency. "Federico II" University, Naples - Italy; 2 Department of General Surgery. "U. Parini" Hospital, Aosta - Italy; 3 Department of Diagnostic Imaging and Radiotherapy. "Federico II" University, Naples - Italy; 4 Department of General, Geriatric, Oncologic Surgery and Advanced Technologies. "Federico II" University, Naples - Italy

## Abstract

**Background:**

Gallstone ileus is a rare cause of gastrointestinal obstruction, more frequent in elderly patients, whose treatment is essentially surgical, although some para-surgical and mini-invasive possibilities exist, allowing the solution of such obstructive condition in a completely non-invasive way.

**Description:**

In our study, after reporting two cases of biliary ileus managed by our surgical division, we will analyze the most suitable diagnostic procedures and the therapeutic approaches to this pathology.

**Conclusions:**

Gallstone ileus is a quite rare pathology in population, but affects more frequently elderly people; The treatment of this disease is mainly surgical.

## Background

Gallstone ileus, the intestinal obstruction due to the migration of gallstones into the intestine lumen, is a quite rare occurrence that must be taken into account in the differential diagnosis of mechanical intestinal obstructions, mainly those affecting small intestine and particularily in elderly patients. The diagnosis of this disease is often late, sometimes detected only during surgery, even if the current routine use of echography and, above all, of TC (more accurate) for abdominal emergencies, allows to detect such condition earlier.

The treatment is basically surgical and the kind of surgery must be chosen according to the risk: a radical surgery in one time should be reserved to selected patients (younger, satisfactory general conditions, absence of serious co-morbidities). The practice of only enterolithotomy is preferred in high risk patients, postponing to a second surgery the solution of the biliary-digestive fistula.

## Clinical case 1

R.M., female, 74 years old, HCV+, with ischemic heart disease, came to our department in april 2009, complaining of 15 days of pain to right upper quadrant with nausea and vomit. After a time of apparent well-being by taking painkillers, the patient shows abundant biliary vomit with 38°C fever and pain. At a physical examination the abdomen is treatable but aching in the right upper quadrant and in the pancreatic duodenal area, peristalsis is present.

**Figure 1:**
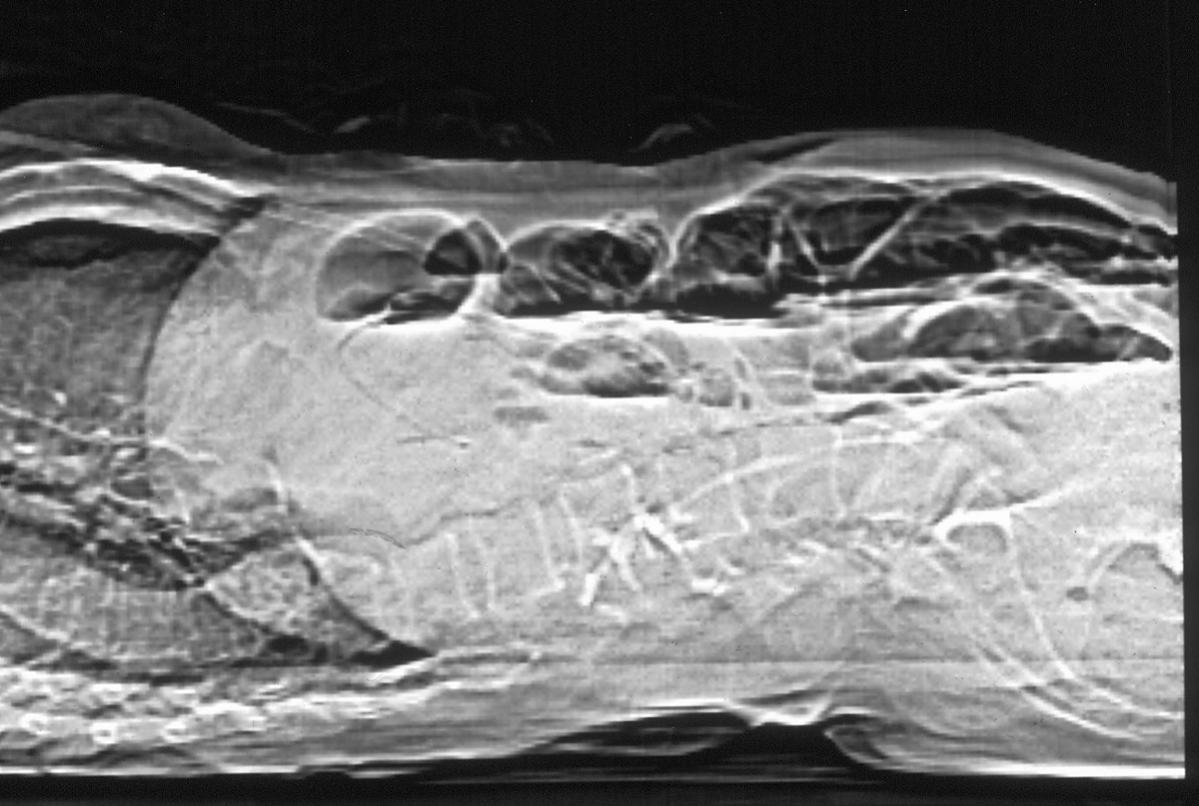
**CT scan L-L**: Marked hydro-gaseous distention of jejunum-ileal loops with multiples air-fluid levels.

**Figure 2:**
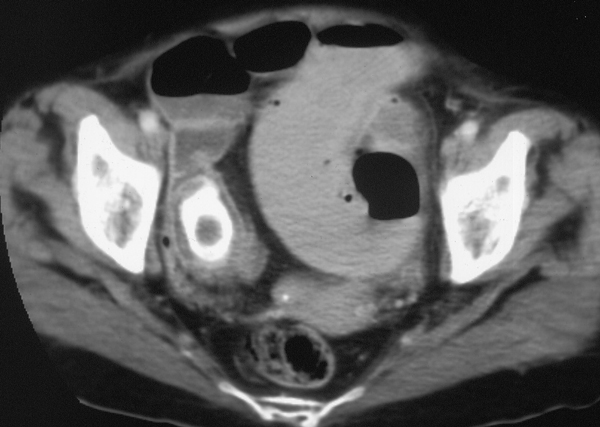
**CT scan case 1**: The gallstone is clearly detectable within the lumen of ileus.

**Figure 3:**
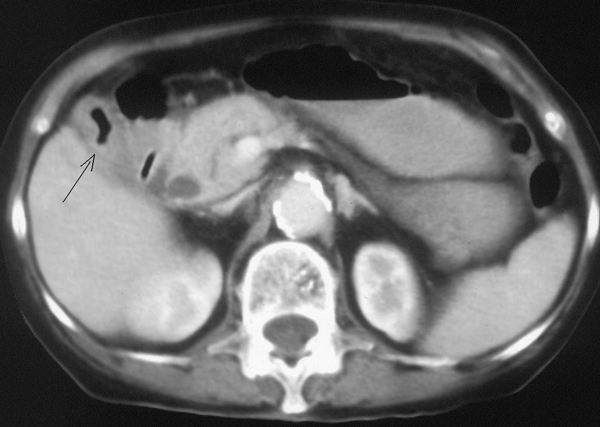
**CT scan case 1**: Gallbladder with thickened walls and with hydrogaseous levels.

Blood test shows low sodium (128 mg/dl), low potassium (2,3 mg/dl), normal values for creatinine and urea, total bilirubin 1,3 mg/dl, 8000 leukocytes; the remaining values, including the most common tumor markers, are normal. TC with contrast to abdomen and pelvis, performed in emergency, shows a marked hydrogaseous distension of jejunum-ileal loops (Figure 1) extended distally up to the terminal ileum where a coarse endoluminal mass (max diam. 2,5 mm) can be detected (Figure 2), with calcium density, multilayered, that indicates a gallstone; it is also present hard gallbladder with thickened walls (Figure 3). The clinical - radiological situation of the patient indicates an intestine obstruction due to ileus gallstone; the patient is taken to the surgery theatre for the emergency laparotomy. A pararectal right-side incision is made; once the peritoneum is opened, many adhesions around gallbladder are visible and we proceed with the adhesiolysis and with the exploration of abdominal organs, that are free of neoplastic lesions. We identify a gallbladder-duodenal fistula, a diverticulum at about 70 cm from the ileocecal valve and a large gallstone formation at ileocecal valve level; we proceed with the enterotomy, with gallstone removal and diverticulum resection with TA. The post surgery course is normal, without complications, with regular discharge from hospital on day 13 after surgery. The patient enjoyed good health for one year and, after 20 months from the surgery, died because of heart disease.

## Clinical case 2

**Figure 4:**
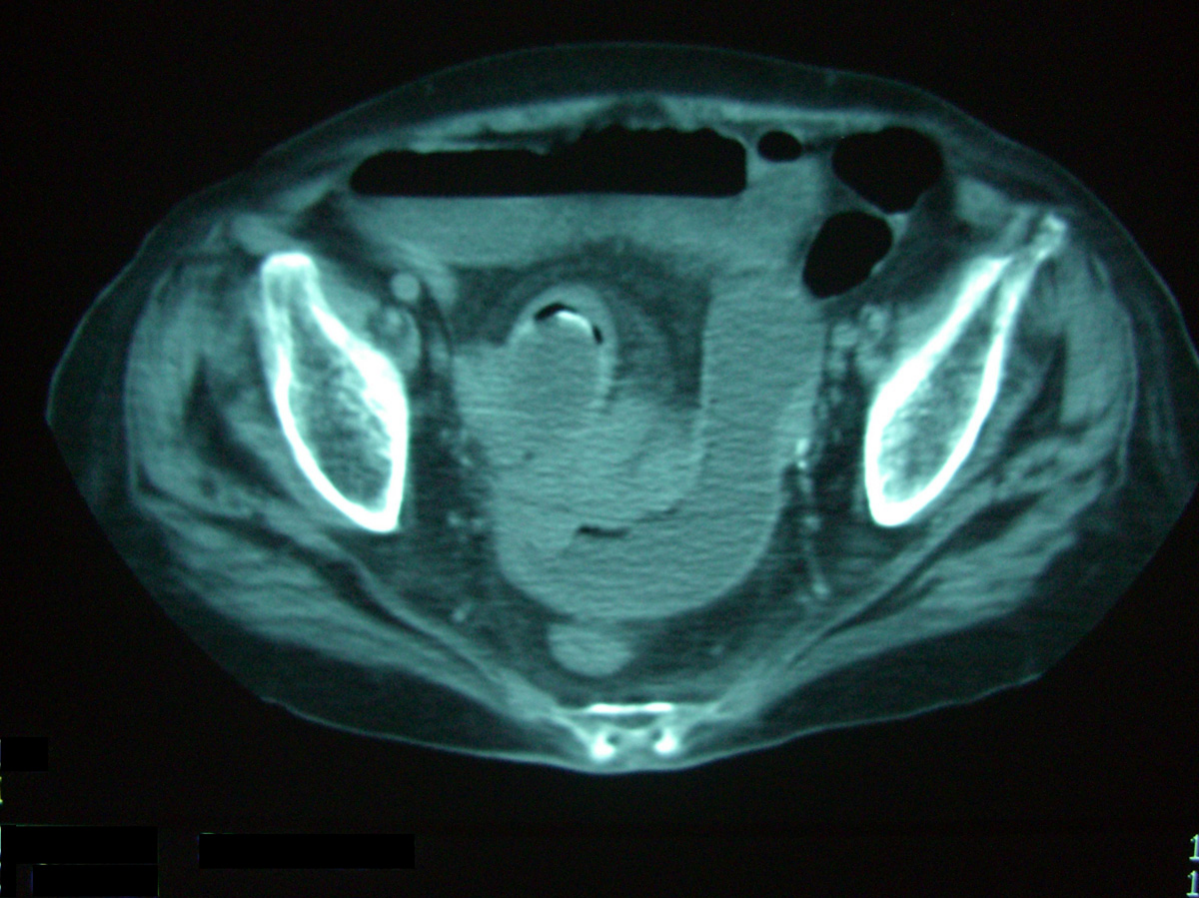
**CT scan case 2**: Intestin loops distention, with air-fluid levels and endoluminal gallstone.

On december 2003 A.T., 83 years old, came to our department complaining of biliary vomit, bloating and pain to the abdomen epi-mesogastric region. At physical examination, abdomen is aching when both deep and surface palpation to upper quadrants are carried out, and some pings (metallic noises) can be heard on auscultation. Blood test does not show significant alterations. An x-ray of the abdomen is performed in urgency and highlights the presence of multiple air-fluid levels; in order to achieve a better knowledge of the patient's situation, we decide to perform abdomen and pelvis TC with contrast which shows the classic triad of gallstone ileus: hard gallbladder, intestine loops distension and air-fluid levels, presence of gallstones in jejunum loops lumen (Figure 4). We decide for urgent surgery; through midline laparotomy we explore the peritoneal cavity and find the dilatation of jejunum-ileal loops that is due to an obstructing gallstone located approximately in the middle of ileum.We proceed cautiously to a mobilization in cranial direction and to ileotomy in healthy bowels, gallstone extraction and following ileotomy suture with an absorbable thread 2/0 single stitches suture. During liver area exploration are found strong gallbladder - duodenum adhesions, but further manipulations are avoided taking into account patient's age and general conditions, and a check of haemostasis and suture of abdomen layered wall is carried out. After 7 days of after-surgery hospitalization without complications, the patient is discharged in a good general condition.

## Discussion

Gallstone ileus causes about 1-4% of intestine obstruction in population [1,2]; such percentage rises to almost 25% in patients older than 65 [3], being the third cause of obstruction after bridles and strangulated hernia. This data highlights the importance of age factor in this pathology: 70% of patients is over 65[4]. Another feature is the higher frequency in females: the average rate is 3,5:1 [5].The gallstone ileus symptoms have, at least at the beginning, their most peculiar expression in the "migrating pain", which is due to what is called "rolling phenomenon": the gallstone, driven by peristalsis, stops and then restarts its migration to a more distal section of intestine, stopping again and so causing the symptoms of an intermittent and discontinuous intestine obstruction [6]. The real disease beginning is at the occurrence of a very strong colic, localized in the right hypocondrium and epigastrium, which denounces the formation of a biliary-digestive fistula [7]; such symptoms tend to attenuate even up to their complete disappearing, but this is actually only momentary (from few hours to some days), after which arise repeated painful crises of the occlusive kind. On the contrary, if the gallstone is small, it can be expelled with no harm to the patient, leaving a biliary-digestive fistula that can remain unknown. Since it is quite difficult to recognize from clinical perspective this particular kind of intestine obstruction, which is often polymorphous, showing insidious onset, underestimated in patients already suffering from biliary colic, x-rays scan is very important (abdomen direct, ecography, TC), like endoscopic and laparoscopic tests which supply a way of diagnosis as well as therapy [8]. Gallstone ileus x-rays diagnostics is based essentially on the Rigler triad [9]: intestine loops dilatation and air-fluid levels (mainly if migrating, fluctuating and changing appearance) sign of intestine obstruction, aerobilia, visualization of a radiopaque image due to a gallstone mass in atypical position, position that can change during the following days. Such triad, actually, is present in less than a half of patients affected by gallstone ileus [10], and this makes infrequent a diagnosis after only x-rays to the abdomen [11]. Ecography is more sensitive than x-rays, since it can show the complete Rigler triad even when abdomen direct shows only the occlusion signs [12]; ultrasonography findings are made of: absent visualization of the gallbladder or presence of hyperechoic foci with posterior acoustic shadowing in the gallbladder bed, aerobilia, intralumen hyperechoic image with posterior acoustic shadowing (gallstone obstructing intestine lumen), image of intestine loops dilatation. TC has proved to be also for this pathology very sensitive and specific, showing the classical signs of Rigler triad even when neither x-rays, nor ultrasound scan could show them [13,14]. Besides the non-invasive techniques, endoscopy is very useful in diagnostics and treatment since it allows not only to detect biliary-digestive fistula, its position, the kind of tissue it is made of, but also to carry out a biopsy that can provide information about the neoplastic nature of the fistula [15]. In addition to this useful information for the diagnosis, endoscopy allows also the treatment of those gallstones that stopped at duodenum and colon level [16], either by extraction with the Dormia drum, or by breaking previously the gallstone with the lithotripter and then extracting the fragments [17]. Laparoscopy proved to be also very useful in the diagnostics of abdominal emergencies, allowing sometimes their treatment [18]. The prognosis of gallstone ileus, if it is not treated by surgery, is fatal; the recovery hope lays exclusively on surgery, which is not free of risk itself. Surgical treatment has some para-surgical and mini invasive alternatives, allowing the solution of such obstructive condition in a totally non invasive way [19]. Surgery performance is divided into two times: one is mandatory and is aimed to the intestine obstruction elimination, the other is not always possible, sometimes not advisable for opportunity reasons, and is aimed to the solution of the biliary-digestive fistula [1,20]. The first phase requires the search of the gallstone nested into the intestine and its extraction by enterotomy (to be carried out on healthy tissue, some centimeters from gallstone impact site, on the anti-mesenteric side, with longitudinal incision); in rare cases it is necessary a segmental resection of the intestinal tract due to the presence of necrosis and perforations. The second phase of surgery is aimed to the solution of biliary-digestive fistula, of the eventual residual gallstones, and final cholecistectomy; this is exactly the most discussed problem: whether to treat contemporarily both the obstruction and biliary fistula (one time treatment), or whether to treat the obstruction only and delay the fistula correction to a second time (two times treatment), or whether to treat the obstruction only, not correcting the fistula at all. Many studies [21,22] have analyzed the pros and cons of such different approaches and the conclusion was that:

• only carefully selected patients with low co-morbidity and good general conditions can stand a long lasting anesthesia and a wide dissection of surgery in one time;

• in case the patient treated with enterotomy only shows symptoms referable to the biliary tract and that cannot be treated differently, cholecistectomy is strongly advisable;

• considering the excellent tolerability of the biliary-digestive fistulas that are not spontaneously closed (event occurring 10% of cases) and the advantages of a treatment with only enterotomy, this seems to be the most suitable surgical treatment for gallstone ileus patients.

The most frequent complication is the wound infection [1,21,22] which occurs to about 30% of patients; the recurrence of gallstone ileus is an always pending risk, that's why abdomen exploration must always be accurate and complete (since recurrence is mainly due to the presence of multiple gallstones upstream or downstream the place where enterotomy is made). The appropriate use of endoscopy (alone or associated with mechanical lithotripsy or shock wave), of extracorporeal shock wave lithotripsy, and of laparoscopy are the new approaches to the treatment of gallstone ileus; such methods are sometimes alternative actions, sometimes first choice, with reference mainly to the patient's condition, to the gallstone ileus shape, to the possibility of using them in a short span of time and, if necessary, repeat them. Endoscopy treatment is possible when gallstones are positioned at gastro-duodenal level [16] or in colon; sometimes, when the gallstone is big, it is possible to use the mechanical lithotripsy (Endoscopic Mechanical Lithotripsy) [17], or, if the gallstone is too hard so that the mechanical lithotripsy is useless, it is possible to use the electrohydraulic kind (Endoscopic Electrohydraulic Lithotripsy). Sometimes it is possible to use the extracorporeal shock wave lithotripsy (Extracorporeal Shock Wave Lithotripsy) which allows to shatter gallstones so that they get to such dimensions as to be expelled with the feces [23]. This method requires some basic requirements: the gallstone must be visible on ultrasound scan (shock waves are reflected or attenuated by intestine gas, so that they are useless), absence of coagulopathy, absence of anenurysms, calcified vessels or bone tissue on the shock wave path. Collateral effects and complicances are really irrelevant. A further possibility of treatment is eventually given by laparoscopy, which has undoubted advantages due to the reduced occurrence of wound infection and a lower occurrence of respiratory problems; with respect to other treatments, laparoscopy approach allows, if carried out by expert hands, also a radical treatment of the disease, avoiding complications that could arise not correcting the biliary-digestive fistula [24-26].

## Conclusions

Gallstone ileus is a quite rare pathology in population, but affects more frequently elderly people; symptoms have little typical elements so that the diagnosis is often late, even if the routine application of ultrasound scan and TC have reduced a lot such diagnosis delay. The treatment of this disease is mainly surgical even if recently some new therapeutic possibilities have been proposed (endoscopy with or without lithotripsy, laparoscopy, extracorporeal lithotripsy).

## List of abbreviations used

CT: computed tomography; HCV: hepatitis C virus; TA: thoracic - abdominal stapler.

## Competing interests

The authors declare that they have no competing interests.

## Authors' contributions

D.M.: conception and design, interpretation of data, given final approval of the version to be published.

G.L.: critical revision, interpretation of data, given final approval of the version to be published.

F.M: acquisition of data, drafting the manuscript, given final approval of the version to be published.

R.S.: acquisition of data, drafting the manuscript, given final approval of the version to be published.

D.R.: acquisition of data, drafting the manuscript, given final approval of the version to be published.

S.E.: acquisition of data, drafting the manuscript, given final approval of the version to be published.

A.B.: conception and design, critical revision, given final approval of the version to be published.

## Authors' information

DM: Assistant Professor of Surgery at University Federico II of Naples.

GL: MD, PhD, in Surgery at University Federico II of Naples.

FM: MD, PhD, in Surgery at U. Parini Hospital of Aosta

RS: Resident in Surgery at University Federico II of Naples.

DR: Resident in Radiology at University Federico II of Naples.

SE: MD, in Radiology at University Federico II of Naples.

AB: Associate Professor of Surgery at University "Federico II" of Naples.

## Declarations

Funding for this article came from personal funds.

This article has been published as part of *BMC Surgery *Volume 13 Supplement 2, 2013: Proceedings from the 26th National Congress of the Italian Society of Geriatric Surgery. The full contents of the supplement are available online at http://www.biomedcentral.com/bmcsurg/supplements/13/S2
